# Droxinostat sensitizes human colon cancer cells to apoptotic cell death via induction of oxidative stress

**DOI:** 10.1186/s11658-018-0101-5

**Published:** 2018-07-28

**Authors:** Ying Huang, Wuping Yang, Huihong Zeng, Chuan Hu, Yaqiong Zhang, Nanhua Ding, Guangqin Fan, Lijian Shao, Bohai Kuang

**Affiliations:** 1 Jiangxi provincial key laboratory of preventive medicine, Nanchang University, Nanchang, 330006 China; 2 Medical School of Nanchang University, 461 Bayi Road, Nanchang, 330006 Jiangxi China; 3 School of Public Health, Nanchang University, Nanchang, 330006 China

**Keywords:** Droxinostat, HT-29 cells, Apoptosis, ROS

## Abstract

Upregulation of histone acetylation plays a critical role in the dysregulation of transcription. It alters the structure of chromatin, which leads to the onset of cancer. Histone deacetylase inhibitors may therefore be a promising way to limit cancer progression. In this study, we examined the effects of droxinostat on the growth of HT-29 colon cancer cells. Our results show that droxinostat effectively inhibited cell growth and colony-forming ability by inducing cellular apoptosis and ROS production in HT-29 cells. Notably, the apoptotic inhibitor Z-VAD-FMK significantly decreased the levels of cellular apoptosis and the antioxidant γ-tocotrienol (GT3) significantly decreased ROS production induced by droxinostat treatment. Z-VAD-FMK and GT3 also partially reversed the negative growth effects of droxinstat on HT-29 cells. GT3 treatment decreased cellular apoptosis and increased colony-forming ability upon droxinostat administration. Z-VAD-FMK treatment also partially decreased droxinostat-induced ROS production. Our findings suggest that the effects of droxinostat on colon cancer cells are mediated by the induction of oxidative stress and apoptotic cell death.

**Electronic supplementary material:**

The online version of this article (10.1186/s11658-018-0101-5) contains supplementary material, which is available to authorized users.

## Introduction

Colorectal cancer (CRC) is one of the most common malignant tumors of the digestive tract: it is the third most commonly diagnosed cancer and the fourth most common cause of cancer death worldwide [1, 2]. Chemotherapy regimens based on 5-fluorouracil (5-FU) remain the standard treatment for CRC in both adjuvant and advanced disease settings and improves overall survival [3]. However, response rates to 5-FU therapy are between 10 and 20% in the metastatic setting [4]. Resistance to chemotherapy is still a major reason for treatment failure in colon cancer [5]. Thus, novel and efficacious therapeutic agents and strategies are urgently needed for the treatment of colon cancer.

Histone deacetylase inhibitors (HDACIs) were recently identified as a promising new target in cancer therapy. Multiple studies have demonstrated that HDACIs can arrest cell growth, block angiogenesis, and induce differentiation and apoptosis in tumor cells [6]. Histones are typically catalyzed by two enzyme families: histone acetyltransferases (HATs) and histone deacetylases (HDACs). Histone acetylation and deacetylation of lysine residues play an important role in the transcriptional regulation of eukaryotic cells [7, 8]. Subsequent functional inactivation and aberrant gene expression of HAT activity or dysregulation of HDAC activity is reported to contribute to cancer initiation and mediate tumor cell proliferation.

HDACIs are therefore now considered attractive as anticancer drugs. Many HDACIs have been shown to sensitize cells to Fas-mediated apoptosis [9] and several HDACIs can synergize with tumor necrosis factor-related apoptosis-inducing ligand (TRAIL) in many kinds of human cancer but not in normal cells [10]. However, the mechanisms of these interactions may be vary by tumor type and drug, with some requiring deeper investigation. For example, the molecular mechanisms underlying the enhancement of colon cell apoptosis by HDACIs remain elusive.

HDACI-induced apoptosis is one essential part of limiting cancer growth and metastasis [11]. There are two major apoptotic pathways: the extrinsic death receptor-mediated pathway and the intrinsic mitochondria-mediated pathway. The truncated Bid protein accounts for the cross-talk between the two [12, 13]. Activation of the extrinsic death receptor-mediated apoptotic pathway, which involves TRAIL and its corresponding cell surface death receptors, leads to the formation of the death-inducing signaling complex (DISC). This event is followed by the activation of caspase-8 [14–16]. Cellular FADD-like IL-1β-converting enzyme-inhibitory protein (c-FLIP) is an important component of DISC with the ability to inhibit the activation of caspase 8 [17, 18]. The mitochondria-mediated pathway is characterized by the loss of mitochondrial membrane potential and the release of cytochrome c from the mitochondria into the cytoplasm. This can be initiated by Bcl-2 family proteins [19].

It has been reported that HDAC inhibitors, such as droxinostat, vorinostat and tubastatin A, induce cellular apoptosis by decreasing the expression of c-FLIP and the anti-apoptotic gene Bcl-2, and increasing the expression of pro-apoptotic genes, such as Bax.

Droxinostat, developed by Reed et al. [20–22], inhibits the activities of HDAC3, HDAC6 and HDAC8 and displays promising anticancer activity [23]. Recent studies have demonstrated that droxinostat can sensitize cells of several cancer cell lines, including PC-3, DU-145, OVCAR-3, HepG2 and SMMC-7721, to death receptor ligands [22]. Droxinostat-induced cell death is mediated by the induction of cellular apoptosis, including caspase 8-dependant extrinsic apoptosis [23, 24]. On the other hand, several reports have demonstrated that some HDACIs stress tissues and cells by causing overproduction of reactive oxygen species (ROS) to induce cell death [25]. Cancer cells are very sensitive to oxidative stress [26]. Whether droxinostat induces oxidative stress in cancer cells is unknown.

In this study, we re-investigated whether treatment with HDACIs, such as droxinostat, would inhibit the growth of HT-29 human colon cancer cells. Mechanistically, we not only elucidated the apoptotic effects of droxinostat on HT-29 cells but also the overproduction of ROS induced by droxinostat treatment.

## Materials and methods

### Reagents

Droxinostat, tubastatin A and PCI-34051 were purchased from ApexBio. FITC-conjugated Annexin V Apoptosis Detection Kit I was purchased from BD Biosciences. Propidium iodide (PI) was purchased from Sigma-Aldrich. DCFDA was purchased from Life Technologies. Z-VAD-FMK was purchased from Selleckchem. γ-tocotrienol (GT3) was purchased from Sigma-Aldrich.

### Cell lines and cell culture

The human colon cancer cell lines used in this study were purchased from the American Type Culture Collection (ATCC). HT-29 or HCT-116 cells were cultured in RPMI medium 1640 supplemented with 10% heat-inactivated fetal bovine serum (FBS), 5958 mg/l HEPES, 100 U/ml penicillin, and 100 μg/ml streptomycin at 37 °C in a humidified 95% air and 5% CO_2_ incubator.

### Cell survival assay

Cell viability was measured using clonogenic and MTT assays. To determine seeding number, different numbers of cells were seeded in 6-well plates without any treatment. For the clonogenic assay, cells were seeded in 6-well plates in triplicate with three different numbers of cells. The cells were fixed after around ten days, stained with 0.1% crystal violet for ten minutes and counted [27]. Colony formation efficiency is defined as the number of colonies observed/the number of cells plated.

For the MTT assay, HT-29 or HCT-116 cells were seeded in 96-well plates in the presence or absence of eight different concentrations of the treatment chemicals (droxinostat, tubastatin A and PCI-34051) for 48 h. The control cells were supplemented with medium containing DMSO (vehicle control). Following treatment, we performed the MTT assay, which is based on the conversion of MTT to MTT formazan by mitochondrial enzymes.

The effect on cell growth was assessed as a percentage of cell viability, with vehicle-treated cells considered 100% viable. The IC_50_ of each chemical was calculated using GraphPad software (GraphPad Software Inc.).

### HDAC3 knockdown

Cultured HT-29 cells in 6-well plates were transfected with: 5 nM HDAC3 on-target plus HDAC3 siRNA-Smart (Ribobio); or 5 nM HDAC3 on-target plus control siRNA using RNAi transfection reagent (Ribobio). The cells were assayed 48 h post-transfection.

### ROS assay and antioxidant treatment

The levels of ROS were measured using flow cytometry with the probe 2′,7′-dichlorodihydrofluorescein diacetate (DCFDA). Briefly, cells were seeded in 6-well plates, incubated overnight and then treated with 21 μM droxinostat for 12 or 24 h. The cells were then detached, centrifuged for 5 min at 200 x g, and stained in 1 ml of DCFDA staining solution (final concentration of DCFDA is 10 μM) or MitoSOX Red (5 μM) at 37 °C for 30 min. The stained cells were washed with 3 ml ice-cold phosphate-buffered saline (PBS) and suspended in 200 μl of PBS. Dead cells were excluded by PI staining. The ROS level was measured using flow cytometry and presented as the mean fluorescence intensity (MFI) of DCF or MitoSOX Red.

For antioxidant effects, cells were seeded in 6-well plates, incubated overnight, treated with 10 μM GT3 for 2 h, and incubated with 21 μM droxinostat for 24 h. The ROS level was also measured via DCFDA using flow cytometry as indicated above.

### Apoptotic assay and anti-apoptotic treatment

Cells seeded in 6-well plates were treated with 21 μM of droxinostat for 12 or 24 h. Following incubation, cellular apoptosis was measured using annexin V and PI staining with an FITC Annexin V Apoptosis Detection Kit (BD Biosciences) following the manufacturer’s instructions.

For the anti-apoptotic effects, cells were treated with 10 μM Z-VAD-FMK at the manufacturer’s recommended concentration for 2 h, and then incubated with 21 μM of droxinostat for 24 h. Following incubation, cellular apoptosis was assessed by annexin V and PI staining following the manufacturer’s instructions.

### Quantitative RT-PCR

Total RNA was extracted from HT-29 cells using the Qiagen RNeasy Mini Kit according to the manufacturer’s instructions. RNA yield and quality were determined by measuring absorbencies at 260 nm and 280 nm, respectively. First-strand cDNA was synthesized in a final volume of 20 μl using the Superscript III First-Strand Synthesis System (Invitrogen). Quantitative RT-PCR analyses were performed using a SYBR Green mix on an ABI StepOne Plus Real-Time PCR System (Applied Biosystems). GAPDH transcripts were used as a housekeeping internal reference for mRNA. The expressions of catalase, superoxide dismutase 1 (SOD1), SOD2 and FLIP-L, Bcl-2, Bcl-xl, BAK, BAX and Puma were calculated using the comparative C_T_ method. The sequences for all the primers used in the quantitative RT-PCR assays are available upon request.

### Caspase-3 activity measurement

The activity of caspase-3 was measured according to the manufacturer’s instructions (Abcam). Briefly, HT-29 cells were suspended in chilled cell lysis buffer after different treatments. Cell lysates were mixed with reaction buffer and DEVD-p-NA substrate for 1 h at 37 °C. The activity of caspase-3 was measured on a microplate reader at OD 405 nm and expressed as the optical density (OD).

### H2AX immunostaining

Cells were fixed with 4% PFA 6 h after incubation with droxinostat. After blocking with 1% bovine serum albumin (BSA), the cells were incubated with anti-phospho-Histone H2AX (1:100, Ser139, Cell Signaling Technology) overnight at 4 °C. After a wash, the cells were stained with Alex 488-conjugated secondary antibody and counterstained with DAPI. The stained cells were viewed and acquired using a Zeiss Axio Observer Z1 microscope (Carl Zeiss Microimaging Inc.) with an Apo 60X/1.4 oil DICIII objective. The images were captured by using AxioVision (4.7.1.0) software (Carl Zeiss).

### Statistical analysis

The data were analyzed with one-way or two-way ANOVA with SPSS statistic 17.0 (SPSS Software) and GraphPad Prism 5 (GraphPad Software Inc.). *p* < 0.05 was considered statistically significant.

## Results

### Droxinostat inhibited the growth of human colon cancer cells

**Fig. 1:**
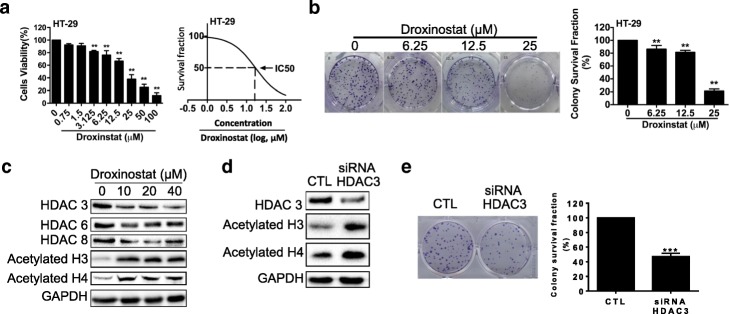
Effects of droxinostat on cell viability in colon cancer cells. **a** – HT-29 cells were treated with the indicated concentrations of droxinostat. The viability of the cells was determined using the MTT assay (left panel). The IC_50_ of droxinostat was calculated using GraphPad software (right panel). Each point represents the mean ± SD of three independent experiments. **b** – Droxinostat with the indicated concentrations was assessed using a clonogenic assay with 500 cells in each well. The significance was determined using one-way ANOVA. **c** – HT-29 cells were incubated with droxinostat for 48 h. The indicated protein was detected via western blot. **d** – Knockdown HDAC3 by siRNA in HT-29 cells. **e** – A clonogenic assay using HDAC3 knockdown cells with 250 cells in each well. **p < 0.01 vs vehicle or control

To estimate the effects of droxinostat on the growth of colon cancer cells, 400 HT-29 cells were seeded onto a 96-well plate and treated with different concentrations of droxinostat. Three days later, the MTT assay was used to measure cell numbers in each dose. The data showed that droxinostat treatment inhibited cell growth in a dose-dependent manner starting from 3.125 μM (Fig. 1a). Using the nonlinear regression method, the IC_50_ of droxinostat was about 21 μM.

To establish the seeded numbers of HT-29 cells for colony assay, we plated 100–4000 cells in 6-well plates. As shown in Additional file 1: Figure S1, the plating efficiencies are 41.00% per 100 cells, 45.75% per 200 cells, 54.10% per 500 cells, 47.30% per 1000 cells, 43.55% per 2000 cells and 30.68% per 4000 cells. We therefore seeded 500 cells in 6-well plates to test the effects of different concentrations of droxinostat on clonogenic ability compared to the clonogenic rate in the control group (56.70%). A dose of 6.25 μM of droxinostat modestly decreased the clonogenic efficiency to 51.90%. However, 12.5 and 25 μM of droxinostat significantly decreased the clonogenic rates to 46.20 and 12.07%, respectively (Fig. 1b).

To confirm the inhibition of droxinostat on HDACs, we harvested protein 48 h after treatment with different concentrations of droxinostat. As shown in Fig. 1c, 10 μM of droxinostat efficiently reduced the expression of HDAC3, 6 and 8 compared to treatment with the vehicle alone. Inhibition of HDACs consistently leads to histone acetylation. Droxinostat treatment obviously increased the expression of acetylated histone H3 and H4 compared to vehicle treatment. To mimic the effects of droxinostat on HT-29 cells, we used siRNA to transiently decrease the expression of HDAC3, resulting in increased expression of acetylated histone H3 and H4 (Fig. 1d). The reduction of HDAC3 expression by siRNA significantly decreased the colony-forming ability in HT-29 cells (Fig. 1e). These data indicate that the activity of HDACs plays an important role in colon cancer cell growth.

To investigate whether the negative effect of droxinostat on HT-29 cells is selective among different HDACIs, we tested the effects of tubastatin A and PCI 34051 on HT-29 cells. As shown in Additional file 2: Figure S2A and B, both tubastatin A and PCI 34051 could inhibit cell growth, although the effective concentration of tubastatin A started from 10 μM and that of PCI 34051 from 40 μM. The IC_50_ values of tubastatin A and PCI 34051 were around 22 and 30 μM, respectively. Droxinostat, tubastatin A and PCI 34051 not only inhibit the growth of HT-29 cells but also that of HCT-116 colon cancer cells (Additional file 3: Figure S3A–C). We then used droxinostat to further explore the mechanisms by which droxinostat efficiently inhibits cancer cell growth. Our results indicate that HDACIs can inhibit the growth of colon cancer cells.

### Droxinostat induced apoptosis in human colon cells

**Fig. 2:**
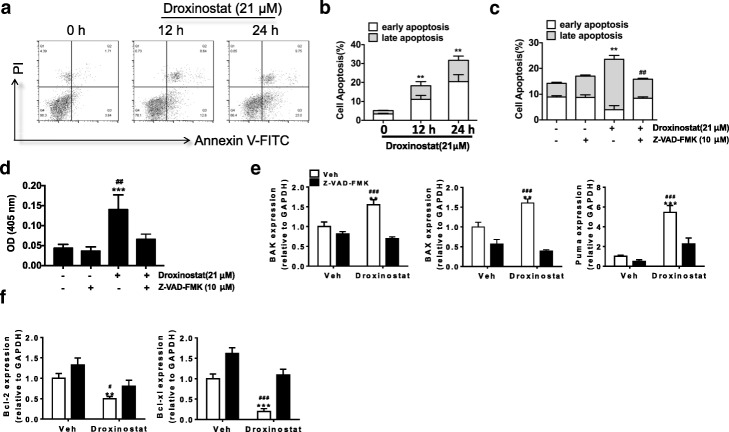
Droxinostat induced apoptosis in colon cancer cells. **a** and **b** – HT-29 cells were treated with droxinostat for 12 and 24 h. Cellular apoptosis was measured with annexin V and PI staining. The percentages of positive cells in the upper right and lower right quadrants represent later and early apoptotic cells, respectively. Data are expressed as the means ± SD of three independent experiments. **p < 0.01 vs vehicle. **c** – HT-29 cells were treated with 10 μM Z-VAD-FMK followed by 21 μM droxinostat treatment for 24 h. Cellular apoptosis was measured using annexin V and PI staining. Apoptotic data are expressed as the means ± SD of three independent experiments. *p < 0.05 vs. vehicle, **p < 0.01 vs. vehicle, ^**##**^*p* < 0.01 vs droxinostat. **d** – HT-29 cells were treated with 10 μM Z-VAD-FMK followed by 21 μM droxinostat treatment for 24 h. The activity of caspase-3 was measured at 405 nm and expressed as optical density (OD). **e** and **f** – HT-29 cells were incubated with droxinostat for 24 h. Expressions of pro- and anti-apoptotic related genes were measured using RT-PCR. **p < 0.01, ***p < 0.001 vs Veh; ^#^*p* < 0.05, ^###^*p* < 0.001 vs droxinostat

Previous studies showed that HDACIs can induce cellular apoptosis, resulting in the inhibition of cancer cell growth [8]. In early apoptosis, membrane phosphatidylserine (PS) translocates from the inner face of the cell membrane to the cell surface. Annexin V can bind to exposed PS with high affinity, whereas PI molecules intercalate inside the DNA double helix in cells with a compromised plasma membrane. We therefore stained cells with annexin V and PI in the absence and presence of droxinostat. As shown in Fig. 2a and b, 21 μM of droxinostat induced apoptosis in up to 21.24% of cells after 12 h treatment (*p* < 0.01) and in 32.75% after 24 h treatment (*p* < 0.01). Notably, the increase in both early and later apoptosis was significant (up to 12.6 and 8.64% at 12 h, and 23.0 and 9.75% at 24 h, respectively) with 21 μM of droxinstat and 12 and 24 h treatment (*p* < 0.01), compared to DMSO treated group.

Z-VAD-FMK is an FMK-based pan caspase inhibitor with superior aqueous stability, cell permeability and efficacy but without cytotoxic effects. To further determine the role of cellular apoptosis in the effects of droxinostat on HT-29 cells, we treated cells with 10 μM Z-VAD-FMK for 2 h prior to droxinostat treatment. Fig. 2c shows that Z-VAD-FMK treatment did not increase cellular apoptosis compared to the DMSO group (*p* > 0.05). Z-VAD-FMK pre-treatment could efficiently inhibit the increase in apoptosis induced by droxinostat from 24.12 to 14.26% with statistical significance (*p* < 0.01). Consistently, droxinostat treatment on HT-29 cells significantly increased the activity of caspase-3, but this effect was reversed by Z-VAD-FMK pre-treatment (Fig. 2d).

Pro- and anti-apoptotic genes play crucial roles during the apoptotic process, so we measured the expressions of apoptosis-related genes with droxinostat and/or Z-VAD-FMK treatment. As shown in Fig. 2e and f, droxinostat treatment increased the expressions of BAK, BAX and Puma while the expressions of Bcl-2 and Bcl-xl decreased with droxinostat treatment. The expression changes of apoptosis-related genes induced by droxinostat were ameliorated by Z-VAD-FMK pre-treatment. These data indicated that droxinostat inhibits the growth of cancer cells, and that this is at least in part due to induction of cellular apoptosis.

### Droxinostat induces oxidative stress in human colon cells

HDACIs induce cellular apoptosis and the formation of reactive oxygen species (ROS). To assess whether the induction of ROS plays a role in the inhibition of HT-29 cell growth upon droxinostat treatment, HT-29 cells were incubated with 21 μM droxinostat or DMSO. After 12 and 24 h, the production of ROS was measured using a DCFDA probe and then analyzed via flow cytometry.

**Fig. 3:**
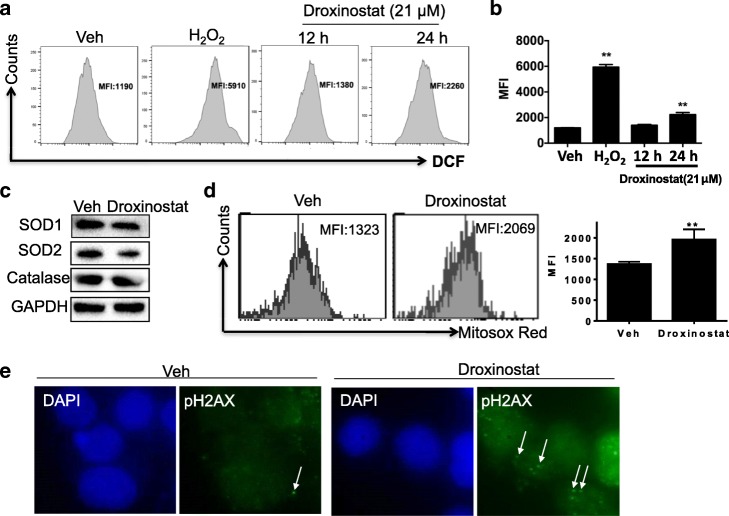
Droxinostat induced oxidative stress in colon cancer cells. **a** and **b** – HT-29 cells were treated with droxinostat for 12 and 24 h. ROS production was measured using the probe DCFDA and analyzed via flow cytometry. The levels of ROS production are expressed as mean fluorescence intensity (MFI) and the means ± SD of three independent experiments. H_2_O_2_ treatment was used as a positive control. **c** – HT-29 cells were incubated with droxinostat for 24 h. The expressions of SOD1, SOD2 and catalase were measured via western blot. GAPDH was used a loading control. **d** – HT-29 cells were incubated with MitoSOX Red 24 h after droxinostat treatment. Representative flow charts are shown, and the MFI is presented. **e** – phospho-Histone H2AX (pH2AX) staining was done 6 h after cells were incubated with droxinostat. DAPI was used to stain the nuclei. Representative pictures are shown (original magnification, 600×). **p < 0.01 vs Veh

As shown in Fig. 3a and b, droxinostat significantly increased the accumulation of ROS in cells (around two-fold) 24 h after treatment (*p* < 0.01). Many antioxidant elements, such as catalase, SOD1 and SOD2, are involved in ROS production. Consistently, droxinostat treatment decreased the expression of catalase, SOD1 and SOD2 when compared to vehicle treatment (Fig. 3c).

MitoSOX Red probe was used to determine whether droxinostat-induced ROS production is derived from the mitochondria. As shown in Fig. 3d, the mean fluorescence intensity of MitoSOX Red was increased (up to 1.5-fold) by droxinostat treatment. These data indicated that droxinostat-induced ROS production is partially derived from the mitochondria. Increased ROS production also causes DNA damage, evidence of which was obtained with phosphorylated H2AX immunostaining (Fig. 3e). These results suggest that droxinostat induces oxidative stress in human colon cells.

**Fig. 4:**
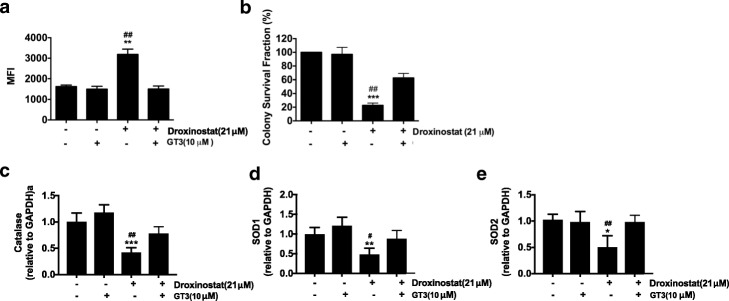
GT3 pretreatment blocked oxidative stress induced by droxinostat treatment in colon cancer cells. **a** – HT-29 cells were treated with GT3 followed by droxinostat treatment for 24 h. ROS production was measured using DCFDA and analyzed via flow cytometry. The levels of ROS are expressed as the means ± SD of three independent experiments. **p < 0.01 vs. vehicle, ^**##**^*p* < 0.01 vs. GT3. **b** – 500 HT-29 cells were seeded in 6-well plates and treated with or without GT3 and droxinostat. Ten days later, the colony numbers were counted and are expressed as the colony survival fraction. ***p < 0.001 vs. vehicle, ^**##**^*p* < 0.01 vs. GT3. C through E – HT-29 cells were treated with GT3 followed by droxinostat treatment for 24 h, and RNA was extracted to measure the expressions of catalase (**c**), SOD1 (**d**) and SOD2 (**e**) via RT-PCR. GADPH was used as a housekeeping control. *p < 0.001 vs. vehicle, **p < 0.001 vs. vehicle, ***p < 0.001 vs. vehicle, ^**#**^*p* < 0.05 vs. GT3, ^**##**^*p* < 0.01 vs. GT3

To further confirm the role of ROS production, we pre-treated cells with 10 μM GT3 for 2 h before droxinostat administration. As shown in Fig. 4a, application of GT3 could partially reduce the increased levels of ROS production after droxinostat treatment (*p* < 0.01). The results from the colongenic assay showed that treatment with GT3 reversed the decreased numbers of colonies induced by droxinostat treatment (Fig. 4b). The expression of catalase, SOD1 and SOD2 was significantly decreased by droxinostat treatment (Fig. 4c–e), and this effect was reversed by GT3 pre-treatment. We concluded that induction of ROS might be one of mechanisms in droxinostat-induced inhibition of cancer cell growth.

### Oxidative stress induced by droxinostat contributes to cellular apoptosis

**Fig. 5:**
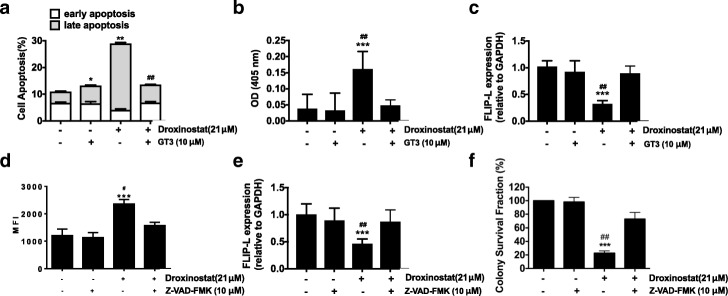
GT3 pre-treatment decreased droxinostat-induced apoptosis in colon cancer cells**. a**– HT-29 cells were treated with 10 μM GT3 followed by 21 μM droxinostat treatment for 24 h. Cellular apoptosis was measured with annexin V and PI staining. Apoptotic data are expressed as the means ± SD of three independent experiments. *p < 0.05 vs. vehicle, **p < 0.01 vs. vehicle, ^**##**^*p* < 0.01 vs. GT3. **b** – HT-29 cells were treated with 10 μM GT3 followed by 21 μM droxinostat treatment for 24 h. The activity of caspase-3 was measured at 405 nm and is expressed as optical density (OD). **c** – HT-29 cells were treated with 10 μM GT3 followed by 21 μM droxinostat treatment for 24 h. Expression of c-FLIP was assessed via RT-PCR. GADPH was used as a housekeeping control. ***p < 0.001 vs. vehicle, ^**##**^*p* < 0.01 vs. GT3. **d** – HT-29 cells were treated with 10 μM Z-VAD-FMK followed by 21 μM droxinostat treatment for 24 h. ROS production was measured using DCFDA and analyzed via flow cytometry. The levels of ROS are expressed as the means ± SD of three independent experiments. ***p < 0.001 vs. vehicle, ^**#**^*p* < 0.05 vs. droxinostat. **e** – HT-29 cells were treated with 10 μM Z-VAD-FMK followed by 21 μM droxinostat treatment for 24 h. Expression of c-FLIP was assessed via RT-PCR. **f** – 500 HT-29 cells were seeded in 6-well plates and treated with or without Z-VAD-FMK and droxinostat. Ten days later, the colony numbers were counted and are expressed as the colony survival fraction. ***p < 0.001 vs. vehicle, ^**##**^*p* < 0.01 vs. Z-VAD-FMK

To further explore the interaction between cellular apoptosis and ROS production induced by droxinostat, the ratios of cellular apoptosis were measured after droxinostat and GT3 treatment. As shown in Fig. 5a, GT3 pre-treatment could efficiently decrease droxinostat-induced apoptosis in HT-29 cells. The increased caspase-3 activity and decreased c-FLIP expression with droxinostat treatment were consistently reversed by GT3 treatment (Fig. 5b and c). These findings indicate that droxinostat induces oxidative stress leading to cellular apoptosis.

Z-VAD-FMK pre-treatment decreased droxinostat-induced ROS production, but the production of ROS induced by droxinostat never returned to normal levels (Fig. 5d). A previous study showed that droxinostat decreased the expression of c-FLIP [28, 29]. As shown in Fig. 5e, c-FLIP expression was indeed reduced by droxinostat treatment. Z-VAD-FMK pre-treatment can prevent the decreased expression of c-FLIP with droxinostat treatment.

Lastly, the colongenic assay showed that the killing effects of droxinostat were reversed by Z-VAD-FMK treatment (Fig. 5f). Therefore, oxidative stress and cellular apoptosis affect each other during droxinostat treatment.

## Discussion

The increased expression and activity of histone deacetylase (HDAC) have been documented in different types of cancers [30]. Dysfunctional HDAC result in normal cell transformation and cancer cell resistance to chemotherapeutic drugs [31]. Therefore, inhibition of HDAC activity has potential as a therapeutic approach to limit cancer cell growth. The development of novel effective HDAC inhibitors (HDACIs) is urgent.

Cells can die of apoptosis through the extrinsic death receptor-induced pathway and/or the intrinsic mitochondrial-mediated pathway [32]. However, programmed cell death is disrupted in cancer, leading to excessive growth of malignant cells [33]. Inducing tumor cell apoptosis is the goal of many cancer therapies.

Our results revealed that droxinostat decreased the expressions of HDAC3, 6 and 8 and increased the expressions of acetylated histone H3 and H4. Droxinostat treatment increased the activity of capcase-3, leading to cellular apoptosis in HT-29 colon cancer cells. Caspase-3 plays crucial roles in both the extrinsic death receptor-induced pathway and the intrinsic mitochondrial apoptotic pathway. The consecutive activation of caspase family members is considered the prerequisite of apoptosis [19]. Furthermore, Z-VAD-FMK, an irreversible pan caspase inhibitor, could inhibitor droxinostat-induced apoptosis in HT-29 cells.

The expression of c-FLIP was decreased by droxinostat treatment in HT-29 cells, which resulted in the activation of the extrinsic apoptotic pathway. Waugh et al. and Zhang et al. also found that HDACIs, such as droxinostat and SAHA, downregulated c-FLIP expression in prostatic cancer cells and hepatocellular carcinoma cells [28, 29].

Previous studies and our data from this study show that HDACIs, including droxinostat, induce cellular apoptosis leading to cancer cell death [28, 34, 35]. A recent report showed that the balance disruption of the anti- and pro-oxidant system might be involved in the cancer cell sensitivity of HDACIs [36–38]. For example, inhibition of HDAC5 increased mitochondrial iron-dependent ROS production in HeLa cells, resulting in apoptosis and autophagy [38]. Panobionstat, a pan HDACI, reduced the viability of HeLa cells through induction of ROS [39]. The data from Lassmann et al. also demonstrated that treatment with HDAC inhibitors negatively regulated the expression of thioredoxin with increasing intracellular oxidative stress and cancer cell death [36, 37].

Our data showed that droxinostat induced oxidative stress and ROS production in colon cancer cells, which is supported by the decreased expression of catalase, SOD1 and SOD2 with droxinostat treatment. γ-tocotrienol (GT3) is a vitamin E analogue with strong anti-oxidant activity, which can decrease the high levels of oxidative stress. Our results indicated that GT3 pre-treatment restored the colony-forming ability affected by droxinostat treatment. This is because GT3 reduces the increased ROS production induced by droxinostat and increases the expression of catalase, SOD1 and SOD2. Notably, GT3 pre-treatment decreased cellular apoptosis, increased c-FLIP expression and reduced the increased caspase-3 activity seen with droxinostat treatment.

Our findings suggest that the increase of ROS production induced by droxinostat in colon cancer cells might further result in apoptotic cell death. Similarly, inhibition of droxinostat-induced apoptosis can partially decrease ROS production under droxinostat treatment.

In summary, we demonstrated that droxinostat effectively reduces the viability of colon cancer cells, and that this is mediated by the induction of oxidative stress and cellular apoptosis. Therefore, droxinostat might have potential as a novel therapeutic agent for the treatment of colon cancer.

## Additional files

Additional file 1: **Figure S1** Colony-forming assay. 100–4000 HT-29 cells were seeded in 6-well plates. The cell culture medium was changed every two days. The colonies were counted ten days after plating (A). Plating efficiency (%) was calculated as the number of colonies observed/the number of cells plated (B). (PPTX 179 kb)

Additional file 2: **Figure S2** Effects of tubastatin and PCI-34051 of cell viability in HT-29 colon cancer cells. HT-29 cells were treated with the indicated concentrations of tubastatin A (A) and PCI-34051 (B). The viability of the cells was determined using the MTT assay. Each point represents the mean ± SD of three independent experiments. The significance was determined using the one-way ANOVA. *p < 0.05 vs. vehicle, **p < 0.01 vs. vehicle. (PPTX 841 kb)

Additional file 3: **Figure S3** Effects of droxinostat, tubastatin and PCI-34051 of cell viability in HCT-116 colon cancer cells**.** HCT-116 cells were treated with the indicated concentrations of droxinostat (A), tubastatin A (B) and PCI-34051 (C). The viability of the cells was determined using the MTT assay. Each point represents the mean ± SD of three independent experiments. The significance was determined using the one-way ANOVA. *p < 0.05 vs. vehicle. (PPTX 76 kb)

## Abbreviations

5-FU: 5-fluorouracil
ANOVA: Analysis of variance
c-FLIP: Cellular FLICE (FADD-like IL-1β-converting enzyme)-inhibitory protein
CRC: Colorectal cancer
DAPI: 4′,6-diamidino-2-phenylindole
DCFDA: 2′,7′-dichlorodihydrofluorescein diacetate
DISC: Death-inducing signaling complex
GT3: γ-tocotrienol
HATs: Histone acetyltransferases
HDACIs: Histone deacetylase inhibitors
HDACs: Histone deacetylases
MFI: Mean fluorescence intensity
MTT: 3-(4, 5-dimethylthiazolyl-2)-2, 5-diphenyltetrazolium bromide
PI: Propidium iodide
PS: Phosphatidylserine
ROS: Reactive oxygen species
SAHA: Vorinostat
siRNA: Small interfering RNA
SOD1: Superoxide dismutase 1
TRAIL: Tumor necrosis factor-related apoptosis-inducing ligand
