# Supplementary figures and images for: Droxinostat sensitizes human colon cancer cells to apoptotic cell death via induction of oxidative stress
Source: Cell Mol Biol Lett. 2018 Jul 28;23:34. doi: 10.1186/s11658-018-0101-5 (PMC6064062; doi:10.1186/s11658-018-0101-5)

## Slide 1
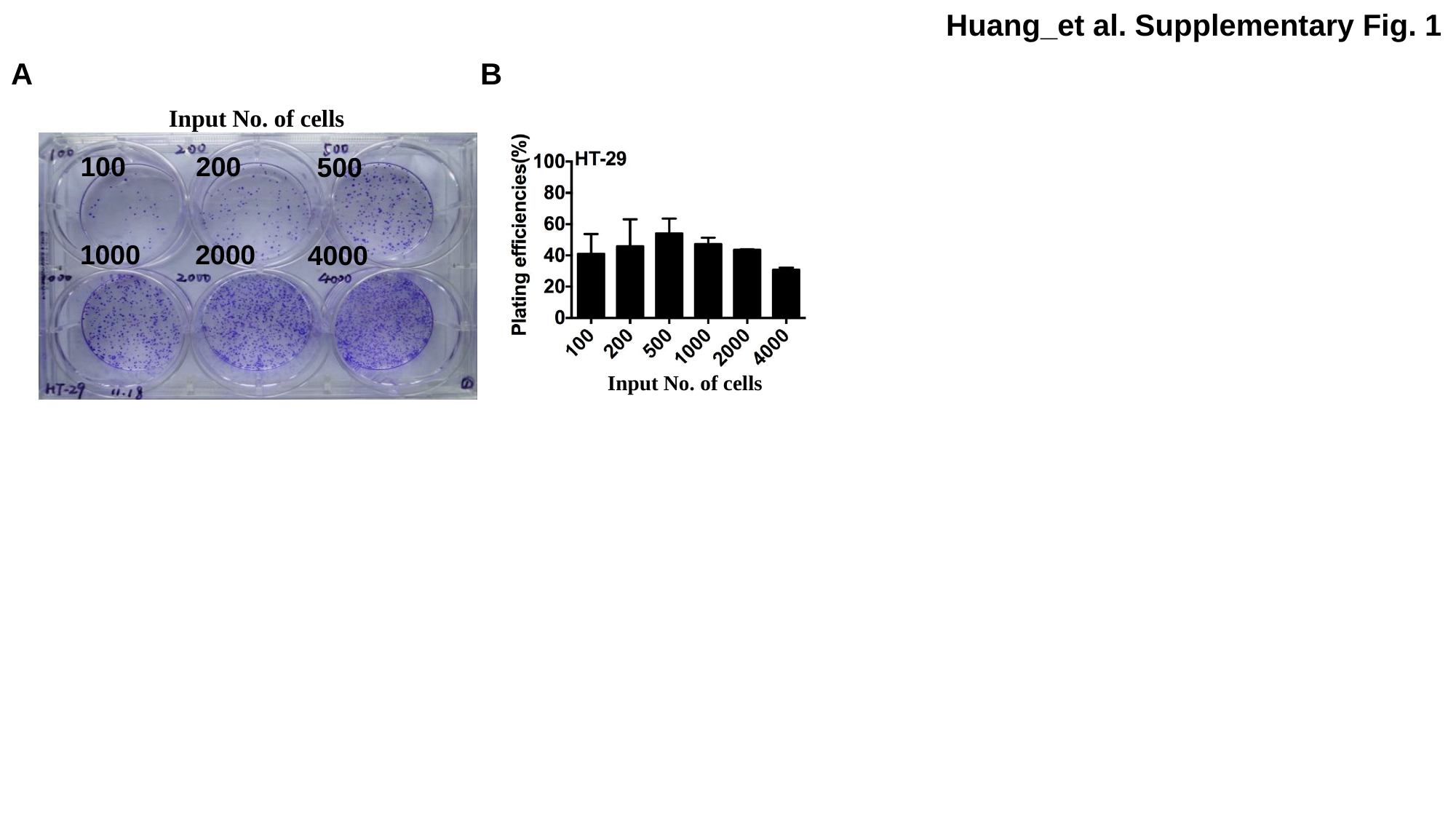

Huang_et al. Supplementary Fig. 1
A
B
Input No. of cells
200
100
500
2000
1000
4000
Input No. of cells

Supplement: Supplementary file 1 — Figure S1 Colony-forming assay. 100–4000 HT-29 cells were seeded in 6-well plates. The cell culture medium was changed every two days. The colonies were counted ten days after plating (A). Plating efficiency (%) was calculated as the number of colonies observed/the number of cells plated (B). (PPTX 179 kb) [file 11658_2018_101_MOESM1_ESM.pptx]

## Slide 1
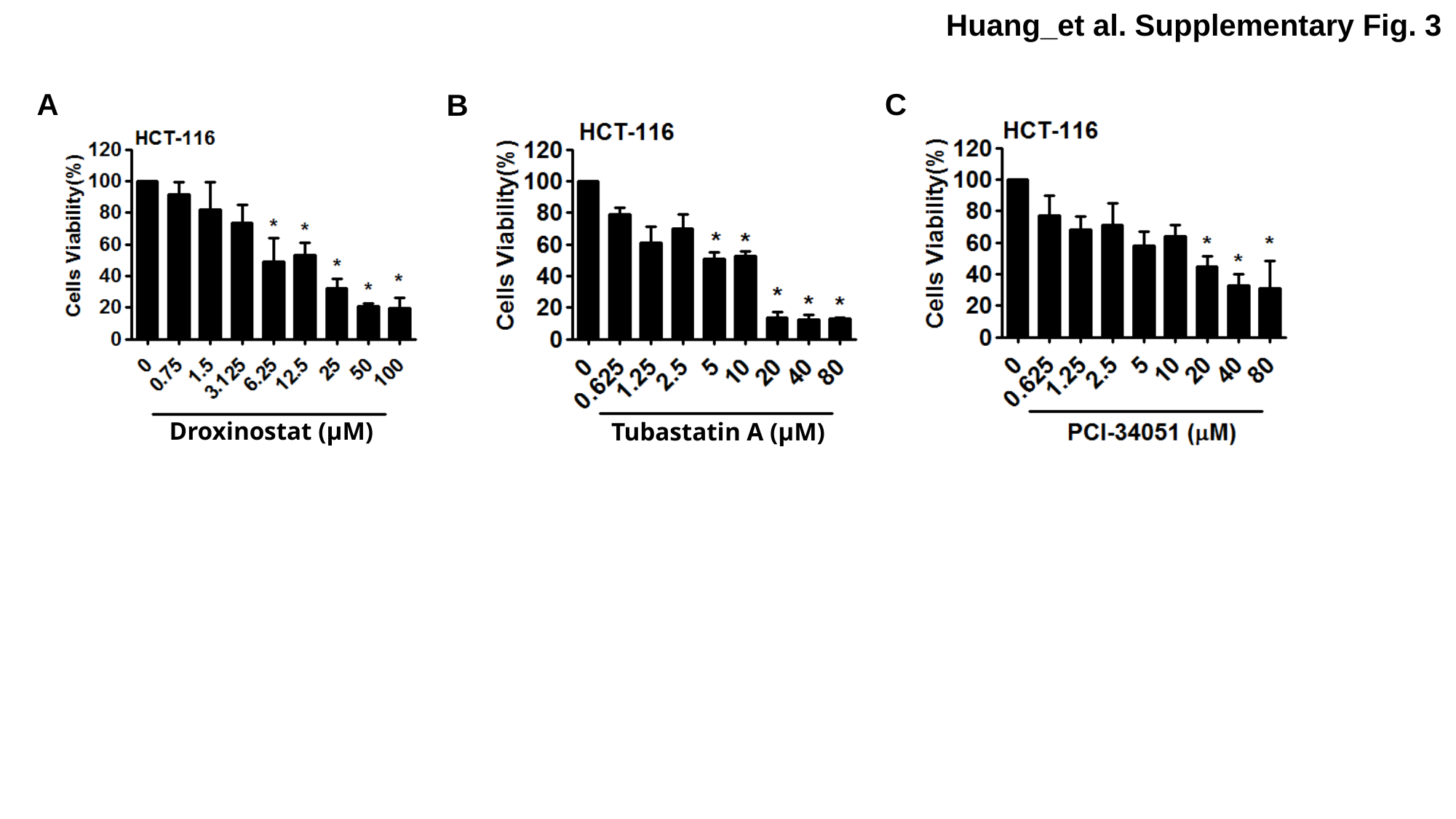

Huang_et al. Supplementary Fig. 3
A
C
B
Droxinostat (µM)
Tubastatin A (µM)

Supplement: Supplementary file 3 — Figure S3 Effects of droxinostat, tubastatin and PCI-34051 of cell viability in HCT-116 colon cancer cells. HCT-116 cells were treated with the indicated concentrations of droxinostat (A), tubastatin A (B) and PCI-34051 (C). The viability of the cells was determined using the MTT assay. Each point represents the mean ± SD of three independent experiments. The significance was determined using the one-way ANOVA. *p < 0.05 vs. vehicle. (PPTX 76 kb) [file 11658_2018_101_MOESM3_ESM.pptx]
