# Supplementary material for: Droxinostat sensitizes human colon cancer cells to apoptotic cell death via induction of oxidative stress
Source: Cell Mol Biol Lett. 2018 Jul 28;23:34. doi: 10.1186/s11658-018-0101-5 (PMC6064062; doi:10.1186/s11658-018-0101-5)
Supplement: Supplementary file 2 — Figure S2 Effects of tubastatin and PCI-34051 of cell viability in HT-29 colon cancer cells. HT-29 cells were treated with the indicated concentrations of tubastatin A (A) and PCI-34051 (B). The viability of the cells was determined using the MTT assay. Each point represents the mean ± SD of three independent experiments. The significance was determined using the one-way ANOVA. *p < 0.05 vs. vehicle, **p < 0.01 vs. vehicle. (PPTX 841 kb) [file 11658_2018_101_MOESM2_ESM.pptx]

## Slide 1
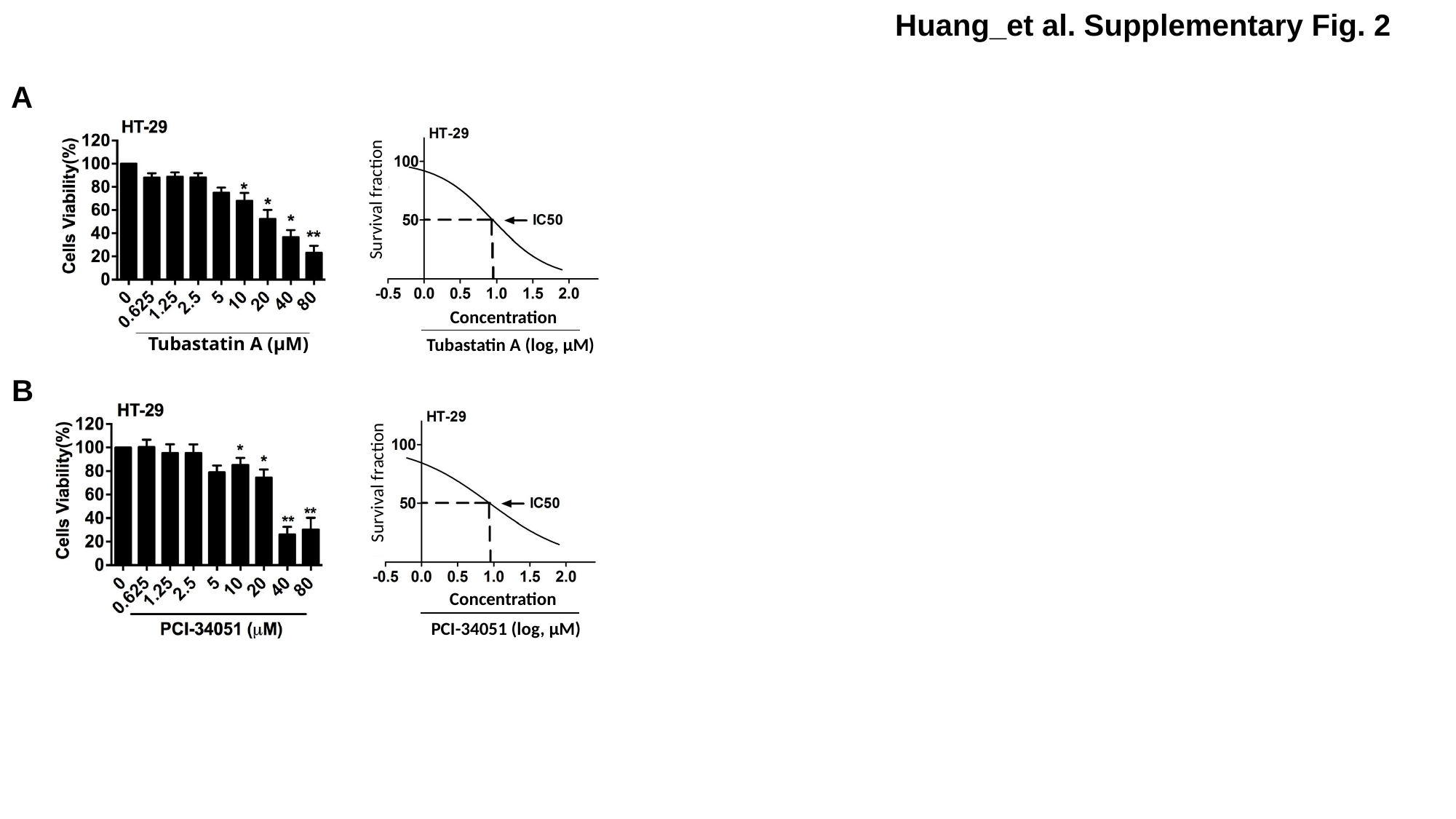

Huang_et al. Supplementary Fig. 2
A
Survival fraction
Concentration
Tubastatin A (µM)
Tubastatin A (log, µM)
B
Survival fraction
Concentration
PCI-34051 (log, µM)
